# Perceived parental alcohol problems and drinking patterns among adolescents in Sweden

**DOI:** 10.1016/j.abrep.2024.100535

**Published:** 2024-02-18

**Authors:** Hiwot Mezgebe Workie, Joakim Wahlström, Johan Svensson, Sara Brolin Låftman

**Affiliations:** a Department of Public Health Sciences, Stockholm University, SE-10691 Stockholm, Sweden; b The Swedish Council for Information on Alcohol and Other Drugs (CAN), Östgötagatan 90, SE-11664 Stockholm, Sweden

**Keywords:** Adolescent drinking patterns, Alcohol, CAST-6, Parental alcohol problems, Sweden

## Abstract

**Introduction:**

Much research into the links between parental problematic alcohol use and adolescent substance use has focused on clinically diagnosed parental alcohol disorders. Few prior studies have utilised validated measures of adolescents’ perception of parental alcohol problems and considered the severity of these problems. This study examined the associations between the severity of perceived parental alcohol problems and adolescents’ drinking patterns in a Swedish national sample.

**Methods:**

We used survey information from grade 9 and 11 students (15–18 years) from 2021 (n = 9,227). Perceived parental alcohol problems were measured by the short version of The Children of Alcoholics Screening Test (CAST-6). The outcomes were: alcohol consumption during the past 12 months, frequent heavy episodic drinking (HED), and early alcohol debut (before age 14). Sociodemographic characteristics were adjusted for.

**Results:**

Binary logistic regressions showed that the severity of perceived parental alcohol problems was associated with alcohol consumption during the past 12 months (low severity OR 1.53, p < 0.001; moderate severity OR 1.85, p < 0.001; high severity OR 2.52, p < 0.001), HED (low severity OR 1.16, n.s.; moderate severity OR 1.31, n.s.; high severity OR 1.64, p < 0.01), and early alcohol debut (low severity OR 1.57, p < 0.001; moderate severity OR 1.65, p < 0.001; high severity OR 2.20, p < 0.001).

**Conclusions:**

Adolescents with perceived parental alcohol problems are more likely to have risky drinking patterns themselves, and the likelihood becomes higher with increased severity. Effective interventions for children whose parents have drinking problems are important, and should also take the severity of the parents’ drinking problem into account.

## Highlights

• Parental alcohol problems were associated with adolescent drinking patterns.
• More severe parental alcohol problems increased the risk of offspring drinking.
• Results were robust also when controlling for sociodemographic characteristics.
• Results were valid for both boys and girls and students in both grades 9 and 11.
• Targeted support crucial to prevent risky drinking patterns in affected adolescents.

## 1 Introduction

The negative consequences of problematic alcohol use extend beyond the individual drinkers and affect others who are directly or indirectly associated with them, including family members, neighbours, co-workers, and the broader community (Rossow et al., 2016a). In previous years, studies examining the adverse effects of problematic alcohol use have primarily focused on the harms and risks experienced by the drinkers themselves. However, in recent years, there has been a shift in focus to alcohol’s harm to others, and particularly on the children of the problematic drinker (Rossow et al., 2016a). Children of parents with problematic alcohol use are considered highly vulnerable to the ‘second-hand effects’ of alcohol as they have limited control over their circumstances and rely on their parents for care and support (Haugland et al., 2021, Jose and Cherayi, 2020). These children face an elevated risk of experiencing adverse social and physical consequences such as neglect, abuse, and dysfunctional family environments (Dube et al., 2001, Haugland et al., 2021, Jose and Cherayi, 2020). They also tend to exhibit higher rates of self-reported health problems (Pisinger et al., 2017, Wahlström et al., 2023, Syed Raza et al., 2023), increased risks of mental and behavioural problems (Johnson and Leff, 1999, Landberg et al., 2019, Raitasalo et al., 2019), and lower educational attainment (Berg et al., 2016, Pisinger et al., 2023). Moreover, children with parents who have alcohol problems are more likely to develop alcohol dependency themselves compared to other children in the same society (Johnson & Leff, 1999). The transmission of alcohol misuse within families is a multifaceted process that involves various pathways and mediators, encompassing both environmental and genetic factors. For example, when parents struggle with alcohol-related issues, it can have an effect on their parenting style and the norms surrounding alcohol use in the household, subsequently impacting their children's alcohol consumption (Latendresse et al., 2008, Ryan et al., 2010). Moreover, Social Learning Theory postulates that children often acquire and emulate their parents' behaviours (Bandura, 1977). Parents may, for example, pass down poor self-regulatory behaviour and impulsivity to their children, thereby exerting influence over offspring drinking patterns (Patock-Peckham et al., 2001, Patock-Peckham and Morgan-Lopez, 2006).

Many studies examining the association between parental problematic drinking and adolescent alcohol use have primarily focused on clinically diagnosed alcohol disorders in parents, disregarding the significant number of parents without clinically diagnosed alcohol problems (Rossow et al., 2016a). However, some studies have explored this association using non-clinical measures of parental alcohol problems. For example, Pisinger et al. (2017) found that secondary education students (mean age of ≈18 years old) in Denmark with perceived parental alcohol problems drank larger quantities of alcohol, had earlier intoxication debut age, and binge drank more frequently than adolescents without perceived parental alcohol problems. Additionally, adolescents who reported being frequently yelled at or often feeling insecure because of parents’ drinking had a higher likelihood of engaging in riskier drinking patterns. A Norwegian study with survey information on alcohol use from both adolescents (mean age of ≈16 years old) and their parents found that hazardous drinking was more common in adolescents with alcohol misusing parents, but that the associations differed based on the gender of both the offspring and the parent (Haugland et al., 2013). Using Swedish cohort data, Thor and colleagues (2022) investigated the link between different levels of fathers’ alcohol consumption and subsequent substance-use disorders in their offspring, tracking this association from adolescence into adulthood. They categorised parental alcohol consumption based on self-reported responses from a questionnaire that the fathers filled out during conscription at ages 18–20 (i.e., well before their (future) children reached adolescence). The categories included abstainers, low or medium level consumers, and those who were frequently intoxicated. The results showed that different levels of parental alcohol consumption imposed different levels of risks for alcohol-related disorders among the offspring. This finding suggests that adolescent alcohol use prevention programs could benefit from considering the severity of the parents' drinking problem. However, one limitation of the study is that it did not account for the fact that alcohol consumption patterns can vary during different life stages, thus not capturing the exact consumption patterns of fathers during the time of their children's upbringing (Thor et al., 2022).

Taken together, although some research exists, it becomes clear that more studies of the links between non-clinical assessments of parental alcohol problems and offspring drinking patterns are needed. In particular, there is a scarcity of research utilising validated measures of adolescents’ perception of parental alcohol problems, that also take the degree of severity into account. The aim of the current study was to examine the associations between the severity of perceived parental alcohol problems and adolescents’ drinking patterns in a Swedish national sample.

## 2 Material and methods

### 2.1 Data material

The data for this study was obtained from the Swedish Council for Information on Alcohol and Other Drugs (CAN) national school survey conducted in 2021. The survey was carried out digitally among students in 9th grade (≈15–16 years of age) and 11th grade (≈17–18 years of age) from a random sample of 350 lower secondary and upper secondary schools across all 21 counties. A total of 9,603 students participated in the survey, with 5,254 students from 9th grade and 4,349 students from 11th grade. The non-response rate at class level was 19 % for grade 9 and 26 % for grade 11, and the non-response rate at the student level was 19 % in both grades (CAN, 2021). For the purposes of this study, we excluded 298 participants who had missing information on the main exposure variable. The majority of these, 196 adolescents, answered none of the questions about perceived parental alcohol problems. Compared to the study sample, those excluded were more likely to have an early alcohol debut age, be male, be in grade 9, not know about their parents’ level of education and not answer or not know about their parents’ country of birth. Additionally, due to survey branching, a number of adolescents did not respond to one or two of the individual outcome variables. Specifically, those who had not consumed alcohol during the past 12 months or never did not answer the questions on heavy episodic drinking and alcohol debut. As a result, the sample size used for analysis varies between 4,665 and 9,227 participants.

### 2.2 Ethics

Since the data from the CAN school survey does not include any personally identifiable information, and the students completed the questionnaire anonymously, formal approval from an ethical review board was not necessary. The participating students provided informed consent. Given the anonymous nature of the questionnaire, the requirement for active written or oral consent did not apply. Additionally, CAN, which was responsible for the data collection, did not seek parental consent, as it is not required for individuals over 15 years of age if they understand what the research means to them (Swedish Ethical Review Act, SFS, 2003:460).

### 2.3 Measures

*Alcohol consumption during the past 12 months* was measured by the question: “Have you ever drunk alcohol in the last 12 months?” with the response categories “No” (0) and “Yes” (1).

*Frequent heavy episodic drinking (HED)* was measured by the question: “Think back to the last 12 months. How often have you, at one and the same occasion, consumed alcohol equivalent to at least four large cans of strong beer/strong cider or 25 cl of spirits or a whole bottle of wine or six large cans of medium-strength beer?” The six response categories were: “Sometimes a week or more often”, “2–3 times a month”, “Once a month”, “2–6 times in the past 12 months”, “Once in the past 12 months”, and “Did not drink in the past 12 months”. Frequent HED was defined as having consumed this amount of alcohol 2–3 times a month or more often (1) vs. others (0). Those who had not consumed alcohol at all during the last 12 months were categorised as missing on this measure.

*Early alcohol debut age* was based on a question about how old the participants were when they had their first glass of alcohol. Early debut age was defined as having at least one glass of alcohol before the age of 14. Those who had not consumed alcohol ever in their life were categorised as missing on this measure.

*Perceived parental alcohol problems* were measured by the short version of The Children of Alcoholics Screening Test (CAST-6), which is a brief screening instrument developed to capture children with problem-drinking parents (Elgán et al., 2021, Ramstedt et al., 2022, Ramstedt et al., 2023, Syed Raza et al., 2023). CAST-6 consists of six items where children are asked to report whether they (1) had felt that their parents drank too much alcohol, (2) wished that their parents stopped drinking, (3) had encouraged parents to stop drinking, (4) had heard parents fight when one parent was drunk, (5) had argued or fought with a parent when the parent was drinking and (6) ever felt like hiding or emptying a parent’s bottle of liquor. Each item includes the term ‘parent(s)’ without specifying whether it pertains to the mother or father. The response categories for all six questions was Yes/No. We followed the procedure by Ramstedt et al. (2023) and created three categories capturing low severity (1–2 affirmative answers), moderate severity (3–4 affirmative answers), and high severity (5–6 affirmative answers) of perceived parental alcohol problems. Internal consistency was high (Cronbach’s alpha 0.87).

Covariates included *gender* (male; female; and other), *grade* (9 and 11), *parental education* (at least one with university education; no parent with university education; and do not know), and *parental country of birth* (at least one parent born in Sweden; two parents born abroad; and do not know) (cf. Syed Raza et al., 2023).

### 2.4 Statistical analysis

We began by examining descriptive statistics for the different samples used in the analyses. Then, we performed further descriptive analyses to examine the proportions of adolescents who reported alcohol consumption during the past 12 months, frequent HED, and early alcohol debut age, respectively, by the severity of perceived parental alcohol problems (ranging between 0 and 6 problems) (cf. Ramstedt et al., 2023). Next, we conducted a series of binary logistic regression analyses to investigate the associations between the severity of perceived parental alcohol problems and each outcome, using the categories low severity (1–2 problems), moderate severity (3–4 problems), and high severity (5–6 problems). No perceived parental alcohol problems served as the reference category. As an additional check we also used CAST-6 as a continuous measure (range 0–6). Unadjusted models included only perceived parental alcohol problems, while adjusted models also included gender, grade, parental education, and parental country of birth. To account for the clustered nature of the data, with students nested within classes, we estimated robust standard errors. Odds ratios and 95 % confidence intervals are presented. Statistical analysis and data processing were conducted by using Stata 17 statistical software (StataCorp, 2021).

## 3 Results

Table 1 presents descriptive statistics for the full sample, used for the analyses of alcohol during the past 12 months (n = 9,227), and for the subsamples used for the analyses of heavy episodic drinking (HED) (n = 4,665), and early alcohol debut (before age 14) (n = 5,274), respectively. Descriptives of the full sample show that among all adolescents, 51.3 % had consumed alcohol in the past 12 months. With regards to perceived parental alcohol problems (CAST-6), 20.5 % reported that they had felt that their parents drank too much alcohol, 13.3 % reported that they wished that their parents stopped drinking, 12.4 % reported that they had encouraged parents to stop drinking, 12.6 % reported that they had heard parents fight when one parent was drunk, 12.2 % reported that they had argued or fought with a parent when the parent was drinking, and 7.7 % responded that they had ever felt like hiding or emptying a parent's bottle of liquor. While 72.0 % did not answer affirmatively to any of these questions on problems with parental drinking, 9.5 % had one affirmative answer; 5.7 % had two; 3.8 % had three; 2.7 % had four; 2.7 % had five; and 3.7 % responded affirmatively to all six items. The study sample consisted of 48.5 % boys, 49.9 % girls, and 1.7 % adolescents who identified as ‘other’ or skipped the question on gender. There was a slight overrepresentation of grade 9 students (54.3 %). The majority of the adolescents (81.3 %), had at least one parent born in Sweden. Lastly, 71.8 % stated that at least one of their parents had attended university education. Of the adolescents who answered the question regarding HED, 11.9 % reported engaging in HED more than twice a month. Regarding alcohol debut age, 19.6 % of those who answered the question started consuming alcohol before the age of 14. The distribution of CAST-6 and the covariates in these subgroups differed slightly compared with the full sample since they only include those who had consumed alcohol during the past 12 months and in their lifetime, respectively. Table A1 in the Appendix displays the distributions of CAST-6 within the full sample and the two subsamples stratified by the dichotomous categories of adolescents’ drinking patterns.

**Table 1:** Descriptives.

	Alcohol consumption during the past 12 months		Frequent heavy episodic drinking (HED)		Early alcohol debut (before age 14)	
	n	%	n	%	n	%
All	9,227	100	4,665	100	5,274	100
**Drinking patterns**						
Alcohol consumption during the past 12 months	4,729	51.3				
Frequent heavy episodic drinking (HED)			553	11.9		
Early alcohol debut (before age 14)					1,033	19.6

**CAST-6**						
Parent drank too much alcohol	1,888	20.5	1,227	26.3	1,370	26.0
Wished that a parent stop drinking alcohol	1,219	13.3	719	15.4	822	15.6
Encouraged one parent to quit drinking alcohol	1,142	12.4	736	15.8	825	15.6
Heard parents fight when one parent was drunk	1,159	12.6	775	16.6	866	16.4
Argued or fought with a parent when the parent was drinking	1,122	12.2	772	16.6	852	16.2
Felt like hiding/emptying a parents' bottle of liquor	706	7.7	467	10.0	524	9.9
*Number of affirmative answers*						
0	6,647	72.0	3,066	65.7	3,475	65.9
1	874	9.5	509	10.9	573	10.9
2	523	5.7	306	6.6	345	6.5
3	349	3.8	215	4.6	248	4.7
4	245	2.7	159	3.4	182	3.5
5	245	2.7	166	3.6	182	3.5
6	344	3.7	244	5.2	269	5.1

**Gender**						
Male	4,475	48.5	2,045	43.8	2,361	44.8
Female	4,602	49.9	2,558	54.8	2,841	53.9
Other	150	1.6	62	1.3	72	1.4

**Grade**						
9	5,000	54.2	1,783	38.2	2,147	40.7
11	4,227	45.8	2,882	61.8	3,127	59.3

**Parental country of birth**						
At least one parent born in Sweden	7,503	81.3	4,161	89.2	4,654	88.2
Two parents born abroad	1,579	17.1	442	9.5	566	10.7
Do not know	145	1.6	62	1.3	54	1.0

**Parental university education**						
At least one parent	6,632	71.9	3,397	72.8	3,792	71.9
No parent	1,123	12.2	586	12.6	682	12.9
Do not know	1,472	16.0	682	14.6	800	15.2

**Fig. 1:**
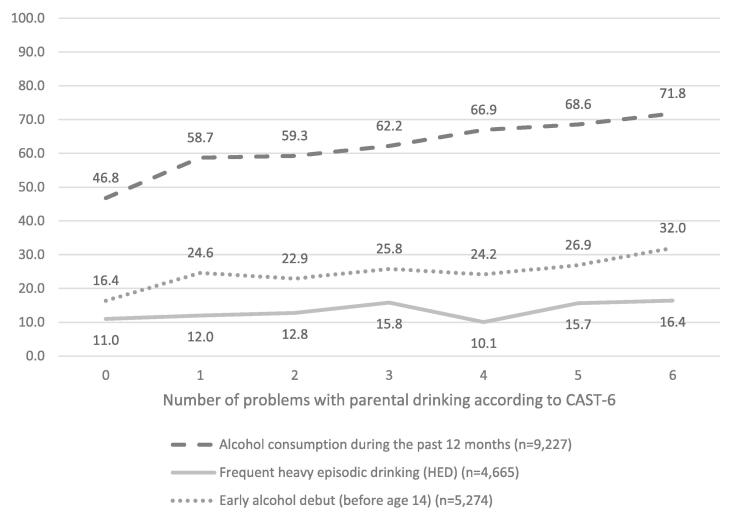
Proportions of adolescents who reported alcohol consumption during the past 12 months, frequent heavy episodic drinking (HED), and early alcohol debut (before age 14) (Y-axis) by number of problems with parental drinking according to CAST-6 (X-axis).

Fig. 1 illustrates the proportions of adolescents who reported the alcohol-related outcomes by the number of problems with parental drinking as measured by CAST-6. The figure shows an increasing trend in the proportions of adolescents who reported alcohol consumption during the past 12 months, frequent HED, and early alcohol debut as the number of reported problems with parental drinking increased. Even among children who experienced only one problem related to their parents' drinking, the proportion was higher compared to those without perceived problem-drinking parents. Notably, the group experiencing all six problems had the largest proportions for each alcohol-related outcome.

The results from the binary logistic regression analyses, examining the associations between the severity of perceived parental alcohol problems and each of the alcohol-related outcomes, are presented in Table 2. In the unadjusted models, it was found that a higher number of problems related to parental drinking, compared with no problems, was associated with an increased likelihood of reporting any alcohol consumption during the past 12 months (low severity: OR 1.63, 95 % CI 1.44–1.84; moderate severity: OR 2.03, 95 % CI 1.73–2.40; high severity: OR 2.71, 95 % CI 2.24–3.29). Similar associations were observed for perceived parental drinking problems and frequent HED, although the estimates for low and moderate severity were not statistically significant (low severity: OR 1.13, 95 % CI 0.88–1.46; moderate severity: OR 1.26, 95 % CI 0.94–1.68; high severity: OR 1.58, 95 % CI 1.18–2.12). For early alcohol debut, there was a graded association between number of perceived problems related to parental drinking (low severity: OR 1.61, 95 % CI 1.36–1.90; moderate severity OR 1.71, 95 % CI 1.34–2.18; high severity OR 2.18, 95 % CI 1.73–2.74). In the fully adjusted models, the associations were similar in strength and remained statistically significant. The crude analyses using CAST-6 as a continuous measure showed positive associations with all three outcomes (alcohol consumption during the past 12 months OR 1.22, 95 % CI 1.18–1.25; HED OR 1.08, 95 % CI 1.03–1.13; early alcohol debut OR 1.15, 95 % CI 1.11–1.19). The estimates were largely similar in the fully adjusted models. Additional analyses stratified by gender and grade were conducted, with similar patterns observed for males and females in both grades 9 and 11 (see Appendix, Tables A2 and A3).

**Table 2:** Drinking patterns among adolescents with low, moderate and high severity in exposure to perceived parental alcohol problems according to a division of CAST-6 into three categories; and by CAST-6 used as a continuous measure. (Reference category = no perceived parental alcohol problems). n = 4,665–9,227.

	Alcohol consumption during the past 12 months (n = 9,227)		Frequent heavy episodic drinking (HED) (n = 4,665)		Early alcohol debut (before age 14) (n = 5,274)	
	Crude	Adjusted	Crude	Adjusted	Crude	Adjusted
	OR (95 % CI)	OR (95 % CI)	OR (95 % CI)	OR (95 % CI)	OR (95 % CI)	OR (95 % CI)
CAST-6						
Low severity (1–2)	1.63*** (1.44–1.84)	1.53*** (1.35–1.74)	1.13 (0.88–1.46)	1.16 (0.90–1.50)	1.61*** (1.36–1.90)	1.57*** (1.32–1.88)
Moderate severity (3–4)	2.03*** (1.73–2.40)	1.85*** (1.54–2.22)	1.25 (0.93–1.67)	1.31 (0.98–1.77)	1.71*** (1.34–2.18)	1.65*** (1.28–2.12)
High severity (5–6)	2.71*** (2.24–3.29)	2.52*** (2.03–3.12)	1.55** (1.16–2.08)	1.64** (1.22–2.22)	2.18*** (1.73–2.74)	2.20*** (1.73–2.81)

CAST-6 continuous measure (0–6)	1.22*** (1.18–1.25)	1.19*** (1.15–1.23)	1.08** (1.03–1.13)	1.09** (1.04–1.14)	1.15*** (1.11–1.19)	1.15*** (1.11–1.20)

*p < 0.05.

** p < 0.01.

*** p < 0.001.

## 4 Discussion

The aim of this study was to investigate the association between the severity of perceived parental alcohol problems and adolescent drinking patterns in a Swedish national sample. The results showed that the severity of perceived parental alcohol problems was linked with more hazardous drinking patterns in a graded manner. This association was observed for alcohol consumption during the past 12 months, frequent heavy episodic drinking (HED), and early alcohol debut. These findings are consistent with previous research studies that have also reported links between perceived parental alcohol problems and adolescent drinking patterns (Alati et al., 2014, Pisinger et al., 2017, Haugland et al., 2013, Sørensen et al., 2011, Thor et al., 2022), and adds to the literature by showing that the severity of perceived parental alcohol problems, as captured by the number of affirmative answers to the questions of the CAST-6 instrument, may play a role. The key result that the severity of perceived parental alcohol problems is important to consider reflects the findings of Thor et al. (2022) who demonstrated graded associations between fathers’ alcohol use and the risk of substance-related disorders in their offspring. Additionally, our findings align with a recent study by Ramstedt et al. (2023), which revealed that the severity of exposure to perceived parental alcohol problems was linked to an increased risk of poor health, impaired social relationships, and problematic school situations among Swedish adolescents.

The mechanisms involved in the familial transmission of parental problematic alcohol use to their offspring have not yet been conclusively determined, as various studies have presented different pathways. Both familial environmental and genetic factors have been proposed as contributing to the development of alcohol use among adolescents with parents who have alcohol problems. Thus, it is evident that understanding the mechanisms of familial transmission of alcohol use is not a straightforward process but rather complex, involving multiple factors and interactions (Karlsson et al., 2016, Schmidt and Tauchmann, 2011, Vermeulen-Smit et al., 2012).

Important family factors that have been discussed in the existing literature include parental care, parental monitoring, and family alcohol norms (Haugland et al., 2013, Van Ryzin et al., 2012, Windle, 1996, Ryan et al., 2010, Latendresse et al., 2008). Parental problematic alcohol use can hinder parents' ability to provide adequate care and nurturing to their children, which are crucial for their physical, mental, and behavioural development (Kuppens et al., 2020). While parental monitoring consistently shows inverse associations with adolescent drinking (Ryan et al., 2010, Mills et al., 2021), parents with problematic alcohol use often exhibit decreased levels of monitoring and discipline towards their children. This may be attributed to their emotional and physical unavailability due to the adverse effects of alcohol, including irritability, hangovers, and mood swings (Windle, 1996). Indeed, Latendresse et al. (2008) and Chassin et al. (1993) have demonstrated that parental discipline and monitoring are essential factors in the association between parental and adolescent alcohol use. Effective parental monitoring involves parents being aware of their children's activities in various domains, including their choice of friends and their actions at home and school (Dishion & McMahon, 1998). In addition, parental monitoring requires good parent–child communication to encourage adolescents to be open to their parents about their activities and for parents to know of any risky behaviour their children may be exposed to, such as substance use (Stattin and Kerr, 2000, Van Ryzin et al., 2012). Parental monitoring can also reduce impulsiveness among offspring, fostering improved control over drinking behaviours and resulting in fewer associated alcohol-related problems (Patock-Peckham et al., 2011). Therefore, low levels of parental monitoring can serve as an environmental mechanism for the transmission of alcohol use from parent to child (Patock-Peckham et al., 2011).

Another possible mechanism for the familial transmission of problematic alcohol use is through parental modelling during the socialisation process. Albert Bandura's Social Learning Theory, which emphasises the influence of observation and imitation on individual behaviours (Bandura, 1977), can serve as a framework in which this transmission can be understood. According to this theory, the family serves as the initial social environment where children learn and develop both risky and healthy behaviours through learning and imitation. Children often learn norms and behaviours by observing and imitating those around them, including their parents' alcohol use (Thor et al., 2022). Contrary to Social Learning Theory that emphasises the imitative transmission of alcohol use, Haller & Chassin (2010) argue there is also a possibility for aversive transmission of alcohol use among children of parents with alcohol problems. Aversive transmission of alcohol use occurs when children with parental alcohol problems choose to abstain from alcohol use due to their observation of the negative consequences that alcohol has imposed on their parents. However, the findings of this study support the argument of Social Learning Theory, indicating that children who grow up in an environment where parents have problematic alcohol use are at a higher risk of engaging in alcohol use themselves.

### 4.1 Strengths and limitations

One strength of this study is the use of a large, national data material collected among adolescents, with perceived parental alcohol problems measured by CAST-6. According to Pisinger et al. (2017), studies on more severe clinical cases may overestimate adverse outcomes, ignoring the numerous non-clinically diagnosed parental alcohol problems in the general population. Another strength is the relatively low non-response rate, implying good possibilities to generalise the findings to the Swedish population of 15–18-year-olds. However, several limitations should be acknowledged. One limitation is that the cross-sectional study design does not enable causal interpretations of the association between perceived parental alcohol problems and adolescent drinking patterns. On the other hand, it seems more plausible that perceived parental alcohol problems impact adolescent drinking patterns rather than the other way around. Notwithstanding, we cannot exclude the possibility that there are omitted variables that may have accounted for the association (e.g., other family psychosocial adversities in the family) (Syed Raza et al., 2023).

It is also relevant to acknowledge systematic bias in the internal non-response to CAST-6 in our data. Participants excluded due to lacking information on CAST-6 (3 % of the total sample) were more prone to having an early alcohol debut, being male, attending grade 9, lacking knowledge about their parents’ education level, and either not providing answers or not knowing their parents’ country of birth. Nonetheless, there are no compelling reasons to believe this bias significantly influenced the observed associations. Another limitation is that while CAST-6 is a lifetime measurement for perceived parental alcohol problems, two of the dependent variables were confined to the last 12 months only, implying potential issues with temporal validity. Relatedly, the temporal order of perceived parental alcohol problems and early alcohol debut age cannot be determined. Further studies that examine the associations between the severity of perceived parental alcohol problems and offspring outcomes based on longitudinal data are warranted. Additionally, while the use of a self-rated measurement of parental alcohol problems is a strength, underreporting of parental alcohol problems may occur as some adolescents may not respond to questions truthfully due to the issue of alcohol disorders being generally taboo (Mackrill et al., 2012). Furthermore, the measure of perceived parental alcohol problems did not allow us to examine if the association with adolescent drinking patterns may differ depending on if it is the mother or the father who has alcohol problems (Haugland et al., 2013); nor if it is one parent or both (Pisinger et al., 2017, Rossow et al., 2016b). Therefore, we recommend future investigations to expand knowledge on these associations.

Lastly, research has also identified genetics as another contributing factor to the familial transmission of alcohol use. Some studies suggest that genetic factors influence the predisposition to alcohol use problems among adolescents with parental alcohol problems (Dick et al., 2011, Verhulst et al., 2015, Agrawal and Lynskey, 2008). Twin and adoption-based studies have provided evidence of genetic heritability in alcohol initiation and problematic alcohol use patterns (Fowler et al., 2007, Poelen et al., 2008). While the current data did not enable analysis of genetic factors, it is crucial for future research to acknowledge the importance of different possible factors in the familial transmission of alcohol use.

## 5 Conclusion

This study showed that adolescents with perceived parental alcohol problems are more likely to have risky drinking patterns themselves, and more specifically, that the likelihood becomes higher with increased severity. These findings highlight the need for effective interventions targeting children who have parents with drinking problems, and emphasise the importance of considering the severity of the parental alcohol problem in designing and implementing such interventions.

## 6 Declaration of Generative AI and AI-assisted technologies in the writing process

During the preparation of this work the authors used Chat GPT in order to improve language clarity and minimise grammatical errors. After using this service, the authors reviewed and edited the content as needed and take full responsibility for the content of the publication.

## CRediT authorship contribution statement

**Hiwot Mezgebe Workie:** Conceptualization, Methodology, Formal analysis, Writing – original draft. **Joakim Wahlström:** Formal analysis, Methodology, Writing – review & editing. **Johan Svensson:** Supervision, Writing – review & editing. **Sara Brolin Låftman:** Conceptualization, Methodology, Writing – review & editing, Supervision.

## Declaration of competing interest

The authors declare that they have no known competing financial interests or personal relationships that could have appeared to influence the work reported in this paper.

## Data Availability

Data will be made available on request.
