# Supplementary material for: Perceived parental alcohol problems and drinking patterns among adolescents in Sweden
Source: Addict Behav Rep. 2024 Feb 18;19:100535. doi: 10.1016/j.abrep.2024.100535 (PMC10900252; doi:10.1016/j.abrep.2024.100535)
Supplement: Supplementary data 1 [file mmc1.docx]

Table A1. CAST-6 by alcohol consumption during the past 12 months, frequent heavy episodic drinking (HED), and early alcohol debut (before age 14). Column per cent.

|  | Alcohol consumption during the past 12 months | | Frequent heavy episodic drinking (HED) | | Early alcohol debut (before age 14) | |
| --- | --- | --- | --- | --- | --- | --- |
|  | No  (n=4,498) | Yes  (n=4,729) | No  (n=4,112) | Yes  (n=553) | No  (n=4,241) | Yes  (n=1,033) |
|  | % | % | % | % | % | % |
| CAST-6 |  |  |  |  |  |  |
| *Number of affirmative answers* |  |  |  |  |  |  |
| 0 | 78.6 | 65.8 | 66.4 | 60.9 | 68.5 | 55.2 |
| 1 | 8.0 | 10.9 | 10.9 | 11.0 | 10.2 | 13.7 |
| 2 | 4.7 | 6.6 | 6.5 | 7.1 | 6.3 | 7.7 |
| 3 | 2.9 | 4.6 | 4.4 | 6.2 | 4.3 | 6.2 |
| 4 | 1.8 | 3.5 | 3.5 | 2.9 | 3.3 | 4.3 |
| 5 | 1.7 | 3.6 | 3.4 | 4.7 | 3.1 | 4.7 |
| 6 | 2.2 | 5.2 | 5.0 | 7.2 | 4.3 | 8.3 |
| Sum | 100 | 100 | 100 | 100 | 100 | 100 |

Table A2. Drinking patterns among adolescents with low, moderate and high severity in exposure to perceived parental alcohol problems according to a division of CAST-6 into three categories; and by CAST-6 used as a continuous measure. (Reference category = no perceived parental alcohol problems). Boys and girls in grade 9.

|  | Boys grade 9 | | | | | |
| --- | --- | --- | --- | --- | --- | --- |
|  | Alcohol consumption during the past 12 months (n=2,474) | | Frequent heavy episodic drinking (HED) (n=744) | | Early alcohol debut (before age 14) (n=952) | |
|  | Crude | Adjusted | Crude | Adjusted | Crude | Adjusted |
|  | OR  (95% CI) | OR  (95% CI) | OR  (95% CI) | OR  (95% CI) | OR  (95% CI) | OR  (95% CI) |
| CAST-6 |  |  |  |  |  |  |
| Low severity  (1-2) | 1.57*** (1.24-1.97) | 1.47**  (1.16-1.86) | 1.68  (0.91-3.08) | 1.70  (0.91-3.16) | 1.59*  (1.10-2.29) | 1.55*  (1.08-2.24) |
| Moderate severity (3-4) | 1.95** (1.28-2.97) | 1.95**  (1.26-3.01) | 0.32  (0.04-2.28) | 0.27  (0.04-1.81) | 1.52  (0.87-2.67) | 1.50  (0.84-2.68) |
| High severity  (5-6) | 2.56*** (1.73-3.79) | 2.56***  (1.67-3.90) | 2.15  (0.89-5.22) | 2.14  (0.83-5.50) | 2.06*  (1.16-3.65) | 2.03*  (1.15-3.59) |
|  |  |  |  |  |  |  |
| CAST-6 continuous measure (0-6) | 1.20***  (1.13-1.27) | 1.20***  (1.12-1.27) | 1.12  (0.96-1.30) | 1.11  (0.97-1.31) | 1.16**  (1.06-1.26) | 1.16**  (1.06-1.26) |
|  | Girls grade 9 | | | | | |
|  | Alcohol consumption during the past 12 months (n=2,423) | | Frequent heavy episodic drinking (HED) (n=1,005) | | Early alcohol debut (before age 14) (n=1,153) | |
|  | Crude | Adjusted | Crude | Adjusted | Crude | Adjusted |
|  | OR  (95% CI) | OR  (95% CI) | OR  (95% CI) | OR  (95% CI) | OR  (95% CI) | OR  (95% CI) |
| CAST-6 |  |  |  |  |  |  |
| Low severity  (1-2) | 1.81***  (1.46-2.25) | 1.61***  (1.28-2.01) | 1.05  (0.55-2.00) | 1.05  (0.55-2.01) | 1.18  (0.85-1.63) | 1.20  (0.86-1.67) |
| Moderate severity (3-4) | 2.47***  (1.84-3.33) | 2.25***  (1.65-3.08) | 1.39  (0.68-2.84) | 1.41  (0.69-2.90) | 1.24  (0.79-1.97) | 1.27  (0.80-2.01) |
| High severity  (5-6) | 3.44***  (2.51-4.70) | 3.13***  (2.28-4.30) | 2.13*  (1.13-4.01) | 2.09*  (1.09-4.01) | 2.13***  (1.40-3.23) | 2.21***  (1.44-3.37) |
|  |  |  |  |  |  |  |
| CAST-6 continuous measure (0-6) | 1.26***  (1.20-1.33) | 1.24***  (1.17-1.30) | 1.13*  (1.01-1.26) | 1.13*  (1.01-1.26) | 1.12**  (1.04-1.20) | 1.13**  (1.05-1.21) |

*p<0.05 **p<0.01 ***p<0.001

Table A3. Drinking patterns among adolescents with low, moderate and high severity in exposure to perceived parental alcohol problems according to a division of CAST-6 into three categories; and by CAST-6 used as a continuous measure. (Reference category = no perceived parental alcohol problems). Boys and girls in grade 11.

|  | Boys grade 11 | | | | | |
| --- | --- | --- | --- | --- | --- | --- |
|  | Alcohol consumption during the past 12 months (n=2,001) | | Frequent heavy episodic drinking (HED) (n=1,301) | | Early alcohol debut (before age 14) (n=1,409) | |
|  | Crude | Adjusted | Crude | Adjusted | Crude | Adjusted |
|  | OR  (95% CI) | OR  (95% CI) | OR  (95% CI) | OR  (95% CI) | OR  (95% CI) | OR  (95% CI) |
| CAST-6 |  |  |  |  |  |  |
| Low severity  (1-2) | 1.54**  (1.16-2.05) | 1.45*  (1.09-1.94) | 1.30  (0.85-1.98) | 1.30  (0.85-2.00) | 1.77**  (1.18-2.65) | 1.77**  (1.17-2.68) |
| Moderate severity (3-4) | 1.61*  (1.08-2.40) | 1.41  (0.93-2.14) | 1.84*  (1.07-3.15) | 1.82*  (1.05-3.14) | 2.29**  (1.30-4.02) | 2.32**  (1.33-4.04) |
| High severity  (5-6) | 2.32**  (1.38-3.91) | 2.12**  (1.25-3.60) | 1.46  (0.77-2.78) | 1.51  (0.79-2.87) | 1.88  (0.98-3.63) | 1.86  (0.96-3.60) |
|  |  |  |  |  |  |  |
| CAST-6 continuous measure (0-6) | 1.18***  (1.09-1.27) | 1.15**  (1.06-1.24) | 1.10*  (1.01-1.19) | 1.10*  (1.01-1.20) | 1.15**  (1.05-1.28) | 1.15**  (1.04-1.27) |
|  | Girls grade 11 | | | | | |
|  | Alcohol consumption during the past 12 months (n=2,179) | | Frequent heavy episodic drinking (HED) (n=1,553) | | Early alcohol debut (before age 14) (n=1,688) | |
|  | Crude | Adjusted | Crude | Adjusted | Crude | Adjusted |
|  | OR  (95% CI) | OR  (95% CI) | OR  (95% CI) | OR  (95% CI) | OR  (95% CI) | OR  (95% CI) |
| CAST-6 |  |  |  |  |  |  |
| Low severity  (1-2) | 1.80***  (1.33-2.44) | 1.57**  (1.16-2.13) | 1.09  (0.72-1.64) | 1.05  (0.70-1.60) | 1.96***  (1.36-2.84) | 1.98***  (1.36-2.87) |
| Moderate severity (3-4) | 1.64*  (1.12-2.38) | 1.47  (0.99-2.18) | 1.28  (0.80-2.04) | 1.27  (0.80-2.04) | 2.12**  (1.36-3.31) | 2.14**  (1.36-3.36) |
| High severity  (5-6) | 2.00**  (1.34-3.00) | 1.91**  (1.27-2.88) | 1.49  (0.94-2.38) | 1.47  (0.91-2.35) | 2.46***  (1.63-3.69) | 2.47***  (1.63-3.74) |
|  |  |  |  |  |  |  |
| CAST-6 continuous measure (0-6) | 1.16***  (1.08-1.25) | 1.14***  (1.07-1.22) | 1.07  (1.00-1.16) | 1.07  (0.99-1.15) | 1.17***  (1.10-1.25) | 1.17***  (1.10-1.25) |

*p<0.05 **p<0.01 ***p<0.001
